# TRIM21 Aggravates Herpes Simplex Virus Epithelial Keratitis by Attenuating STING-IRF3-Mediated Type I Interferon Signaling

**DOI:** 10.3389/fmicb.2020.00703

**Published:** 2020-04-16

**Authors:** Tianchang Tan, Likun Xia

**Affiliations:** Department of Ophthalmology, Shengjing Hospital of China Medical University, Shenyang, China

**Keywords:** herpes simplex virus-1, keratitis, TRIM21, type I interferon, STING

## Abstract

Herpes simplex virus-1 (HSV-1) is the leading cause of infectious blindness in the developed world. HSV-1 infection can occur anywhere in the eye, and the most common presentation is epithelial keratitis. In the HSV epithelial keratitis mice model, we detected the expression of TRIM21 and then investigated the clinical relationship between TRIM21 and HSV epithelial keratitis by silencing TRIM21. Through the clinical scores and histopathology examination, we found that TRIM21 can effectively reduce the severity of HSV epithelial keratitis. Furthermore, silencing TRIM21 significantly controlled the virus particle release at 1, 3, and 5 days post-HSV-1 infection. Notably, the production of IFN-β was enhanced, and the secretion of pro-inflammatory cytokines (IL-6 and TNF-a) was inhibited. Next, human corneal epithelial cells were pretreated with lentivirus or siRNA, respectively, so that TRIM21 expression was overexpressed or silenced. We focused on the regulation of STING-IRF3 and type I interferon signaling after infected with HSV-1. In conclusion, our results have identified that TRIM21 is abnormally high expressed in HSV epithelial keratitis. TRIM21 enhances the replication of HSV-1 in corneal epithelial cells via suppressing the production of type I IFN by inhibiting STING/IRF3 signaling. It also promotes the secretion of IL-6 and TNF-a, thereby aggravating the severity of HSV epithelial keratitis.

## Introduction

Herpes simplex virus-1 (HSV-1) is a neurotropic double-stranded DNA virus, which is the leading cause of infectious blindness in the developed world (Rechenchoski et al., 2017). Epidemiology shows that HSV-1 has been recognized as a ubiquitous human pathogen, infecting 50–90% of the world population (Smith and Robinson, 2002). Eye disease caused by HSV-1 infection usually presents as epithelial keratitis, which accounts for 50–80% of ocular herpes (Labetoulle et al., 2005). Although HSV epithelial keratitis is self-limiting within one week, without adequate treatment, it may progress to stromal keratitis, leading to progressive corneal opacity (Wilhelmus, 2015).

Primary infection is usually caused by direct infection of the cornea by HSV-1. After infection, HSV-1 replicates in the corneal epithelial cells and then triggers innate immune signaling through the production of cytokines and chemokines. Subsequently, immune cells are recruited to the site of primary infection, predominantly neutrophils and dendritic cells, which at this stage function to limit viral spread within corneal epithelial cells (Lobo et al., 2019). At present, antiviral medicines, interferon drops and superficial wiping have been used to cure HSV epithelial keratitis. However, controversies persist about the side effects of antiviral drugs, the effectiveness of topical interferons and the safety of debridement methods (Wilhelmus, 2015). The treatment objective of HSV epithelial keratitis is to inhibit viral replication and to prevent the active viral infection of corneal cells.

Type I interferon is one of the first cytokines secreted upon HSV-1 infection of the cornea, which can limit virus transmission by inhibiting virus replication and increasing the resistance of neighboring cells (Ellermann-Eriksen, 2005). Several studies have shown that type I interferon is necessary for controlling the replication of HSV-1 in the cornea, and it is also required for immune cells to recruit to the site of infection (Hendricks et al., 1991; Leib et al., 1999; Conrady et al., 2011b). Therefore, enhancing the ability of host cells to produce type I interferon may be the best treatment for HSV epithelial keratitis.

The tripartite motif (TRIM) protein family consists of up to 100 members and is the largest group of E3 ubiquitin ligases in mammals (Su et al., 2016). Many TRIMs proteins have been reported to have direct or indirect antiviral activity (Khan et al., 2019). TRIM21, also known as Ro52/SS-A or RNF81, has a B30.2 domain encoded in the C-terminal region, which comprises a combination of a PRY motif followed by a SPRY motif (Rhodes et al., 2005). B30.2 domain can be recruited to incoming viral cores and determines antiviral specificity (Towers, 2007). Recent studies have shown that TRIM21 can recruit proteasomes and savagely break down capsids so that the exogenous viral genomes are exposed prematurely to promote the activity of PRRs (Watkinson et al., 2015). HSV-1 belongs to an enveloped virus. Thus we suspect that TRIM21 may affect HSV-1 infection. In this study, we have investigated the role of TRIM21 in the host defense against HSV-1 infection in a murine model of HSV epithelial keratitis and explored its underlying mechanism in human corneal epithelial (HCE) cells.

## Materials and Methods

### Mice, Cell, and Virus

Female 6-week old C57BL/6J mice were purchased from Liaoning Changsheng Biotechnology Company (Benxi, China) and maintained in the specific pathogen-free environment. All investigations followed guidelines of the Institutional Animal Care and Use Committee of China Medical University and adhered to the ARVO Statement for the Use of Animals in Ophthalmic and Vision Research. During experimental procedures, suffering minimized by isoflurane anesthesia. The HCE cells (BNCC341100) harvested in Dulbecco’s modified Eagle’s medium (DMEM, Hyclone, China) with 10% fetal bovine serum (FBS, Hyclone). The Vero cells (ATCCCCL-81) maintained in Minimal Essential Media (MEM, Hyclone) supplemented with 10% FBS. The cells were maintained at 37°C with 5% CO_2_ in a humidified atmosphere. HSV-1 Mckrae strain was propagated and titrated in Vero cell monolayers, followed by stored in aliquots at −80°C until used as previously described (Stremlau et al., 2004).

### Mouse HSV Epithelial Keratitis Model and Pre-treatment

Development of HSV epithelial keratitis in C57BL/6J mice was studied with HSV-1 McKrae strain and 10^6.6^. TCID_50_/ml of the virus was applied to the right cornea in 5 μl MEM after abrading in a cross-shaped pattern with a sterile 27-gage needle. While the control group was instead received 5 μl MEM, leaving them uninfected. For the pre-treatment, 3 μmol of siRNA-TRIM21 or siRNA-control were subconjunctivally injected into the right eye once a day for three times before establishing HSV epithelial keratitis models. The dosages used in the experiment were evaluated in the pre-experiment.

### Clinical Observation and Scoring

Corneas were infected with HSV-1 and scored the severity of HSV epithelial keratitis by slit-lamp biomicroscopy (Kawa Co., Japan) in a blinded manner every day. The clinical scoring system was as follows (Komoto et al., 2015): 0, entire epithelial area intact; +1, diffuse punctate lesion; +2, dendritic lesion occupying less than 1/4 of the entire epithelial area; +3, severe dendritic lesion extending more than 1/4 of the entire epithelial area; +4, geographic lesion on the epithelial area.

### Virus Titers Assay

Swabs harvested tear samples on the 1, 3, and 5 days after infection. Then all the swabs with tear samples were stocked in 1ml of MEM with 2% FBS and stored at −80°C until used. Titers were determined by TCID_50_ assay on Vero cell monolayers with standard methodology. Briefly, cell culture lysis supernatants were diluted serially using 10-fold dilutions and titered on Vero cell monolayers by the TCID_50_ assay.

### Western Blotting Assay

The total protein from mouse corneas (3 corneas/sample/group) and HCE cells was extracted and prepared in a standardized manner for Western blotting. After SDS-PAGE, transfer and blocking with 5% non-fat milk at room temperature for an hour on a shaker, membranes were incubated overnight at 4°C with anti-TRIM21 antibody (Abcam, United Kingdom, used at 1:1000), STING (D2P2F) Rabbit mAb (Cell Signaling Technology, China, 1:1000), IRF-3 Rabbit mAb (Cell Signaling Technology, 1:1000), phospho-IRF3 Rabbit mAb (Cell Signaling Technology, 1:1000) and GAPDH antibody (Wanlei, China, 1:1000) as primary antibodies. Followed by the membranes were washed three times in Tris-buffered saline. Then, the membranes incubated with goat horseradish peroxidase-conjugated anti-mouse or anti-rabbit antibodies (Beyotime, China, 1:5000) as secondary reagents for 2 h at room temperature. Finally, an enhanced chemiluminescence kit (Wanlei, China) was utilized to visualize the membrane. The densitometry analysis was performed using ImageJ 6.0 software.

## Elisa

Corneas (3 corneas/sample/group) were cut in small pieces and homogenized in 0.5 mL of PBS with 0.1% Tween 20. All samples were centrifuged at 13000 × *g* for 15 min to remove tissue debris. Then, levels of IL-6 and TNF-a in supernatants were detected by ELISA kits according to the manufacturer’s instructions. ELISA kits were all purchased from R&D Systems.

### Quantitative PCR Analysis

Total mRNA from infected corneas and cells were extracted using the RNAiso Plus kit (Takara, Japan), and then stored at -80°C until used. Total mRNA was reverse-transcribed using PrimeScript^TM^ RT reagent Kit with gDNA Eraser to generate cDNA (Takara, Japan). Quantitative PCR (qPCR) was performed using an SYBR Green (Takara, Japan) format with 7500 Fast Real-Time PCR Detection Systems (Life Technology). The sequences of the qPCR primers used in the analyses are summarized in Table 1. The expression levels of different molecules were normalized to GAPDH using ΔCt calculation.

**TABLE 1:** List of primers.

Gene	Forward primer (5′–3′)	Reverse primer (5′–3′)
TRIM21	AGAGAGACTTCACCTGTTCTGT	TCAGTTCCCCTAATGCCACCT
IFN-β	CCCTATGGAGATGACGGAGA	CTGTCTGCTGGTGGAGTTCA
GAPDH	TGGATTTGGACGCATTGGTC	TTTGCACTGGTACGTGTTGAT

### Histopathology and Immunofluorescence Staining

Mice were sacrificed by cervical dislocation at the indicated time point, and then corneas were collected into 1.5 ml of 4% paraformaldehyde for fixation. Paraffin-embedded corneas were then sectioned at 5 μm thickness. The sections were incubated with H&E staining to determine the pathologic changes of corneas. For immunofluorescence staining, the sections were incubated with diluted TRIM21 primary antibody (Santa, United Kingdom, 1:150) overnight at 4°C. After washing thrice with PBS (10 min each time), fluorescence-conjugated secondary antibody (Santa, United Kingdom, 1:50) working solution were added and incubated at 37°C for 2 h. Subsequently, DAPI (Invitrogen, United Kingdom) was added for 5 min to visualize the nuclei. Finally, the fluorescence signal was observed under the fluorescence microscope (Olympus, Japan).

### Statistical Analysis

Three independent experiments were performed for each assay. All results were expressed as mean ± standard deviation and analyzed by Student’s *t*-test. Rank data comparison using the rank-sum test. The statistics are presented using GraphPad Prism five to evaluate the differences. A *p*-value < 0.05 was considered to be statistically significant. All methods were performed following the relevant guidelines and regulations.

## Results

### Expression of TRIM21 in Murine Corneas in Response to HSV-1 Infection

**FIGURE 1:**
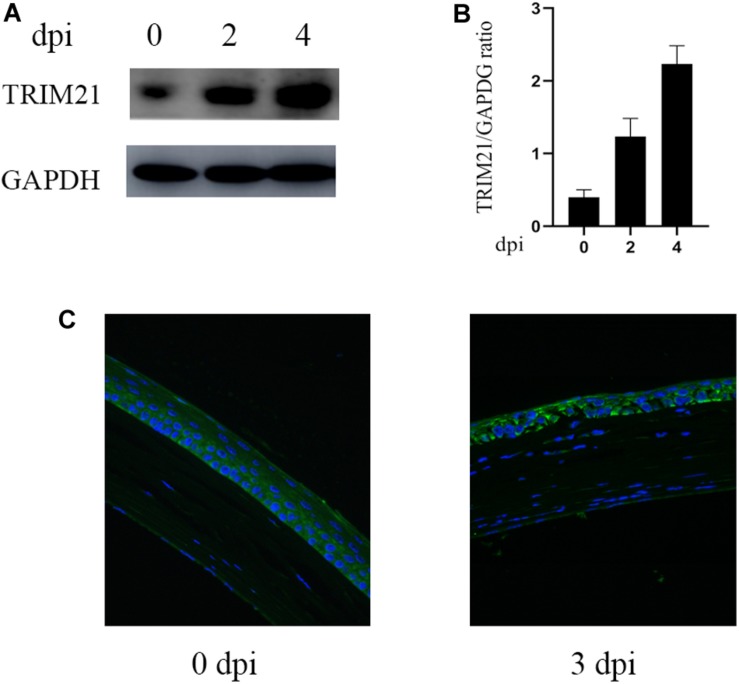
Abnormal expression of TRIM21 in C57BL/6 mouse cornea after HSV-1 infection. **(A)** The protein expression level of TRIM21 was examined by western blotting in corneas at 0, 2, and 4 days post HSV-1 infection (dpi). Three corneas were used per experiment. **(B)** Relative integrated density values quantitated TRIM21 protein levels after normalization to GAPDH. Data were representative of three individual experiments and represented as the mean ± SD. **(C)** At 0 and 3 dpi, the expression of corneal TRIM21 was evaluated by immunofluorescent staining. TRIM21 staining showed green fluorescence, and nuclear staining showed blue fluorescence. Magnification: 400×.

To explore the potential role of TRIM21 in HSV epithelial keratitis, its expression was measured in corneas of epithelial keratitis mice at 0, 2, and 4 days post-HSV-1 infection (dpi). As shown in Figures 1A,B, compared with uninfected corneas, HSV-1 infected corneas displayed abnormally elevated TRIM21 level. Overall, as the course of HSV epithelial keratitis progressed, the expression of TRIM21 continued to increase. To further confirm whether TRIM21 was involved in the pathological process of HSV epithelial keratitis, we detected the localization of TRIM21 in corneas by immunofluorescence, which labeled TRIM21 with green fluorescence (Figure 1C). Results showed that TRIM21 was predominantly expressed in the corneal epithelium at 0 dpi compared with the expression of TRIM21 was increased significantly at 3 dpi. TRIM21 mainly expressed in the cytoplasm of corneal epithelial cells.

### The Contribution of TRIM21 in HSV Epithelial Keratitis

**FIGURE 2:**
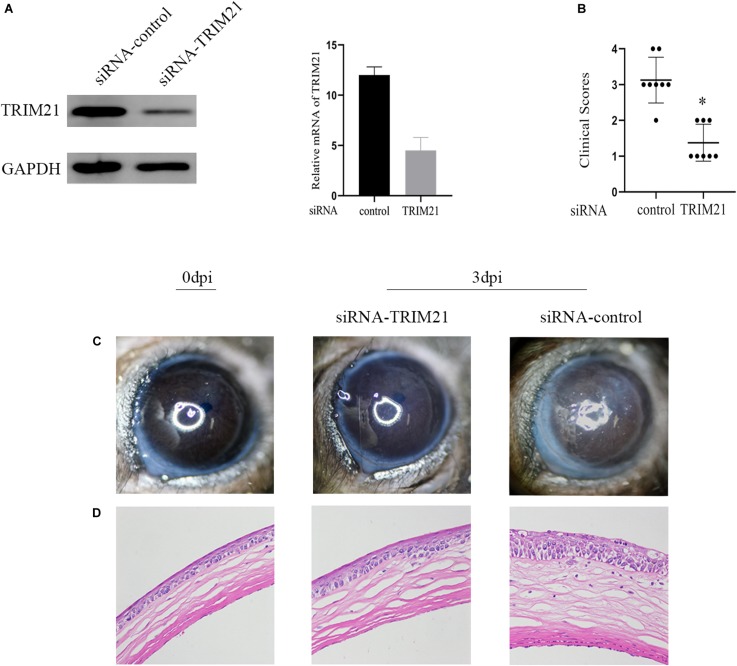
Silencing TRIM21 alleviates the severity of HSV epithelial keratitis. **(A)** The protein (left) and mRNA (right) levels of TRIM21 in corneas were assessed to confirm the transfection efficiency of siRNA before establishing HSV epithelial keratitis models. **(B)** HSV epithelial keratitis clinical scores at 3 dpi. *n* = 8 per time point. **(C)** Representative photographs of corneas from each group of mice at 3 dpi corresponding to **(D)** histopathology results. Magnification: 400×.

To further ascertain the role of TRIM21 in HSV epithelial keratitis, siRNA transfection was used to limit TRIM21 expression in the corneas before establishing the HSV epithelial keratitis mice model. Knockdown efficiency (Figure 2A) was assessed by the protein and mRNA levels of TRIM21 in corneas. After that, corneas were infected with HSV-1 and then examined to score the severity of HSV epithelial keratitis by hand-held slit lamp microscope in a blinded manner every day. We found that the clinical scores and the degree of corneal opacity in the siRNA-TRIM21 group were significantly lower than that in the siRNA-control group (*p* < 0.05, Figures 2B,C). Figure 2B showed that the number of corneal lesion score of ≤2 in the siRNA-TRIM21 group (8/8) was more than the number in the siRNA-control group (1/8). As shown in Figure 2D, compared with histopathology in the siRNA-TRIM21 treated corneas, corneal epithelial cells in the siRNA-control treated corneas showed obvious proliferation and growth disorders. Corneal epithelial layer showed vacuole-like changes, and a small number of inflammatory cells infiltrated into the superficial layer of the corneal stroma. These results indicated that silencing TRIM21 can reduce the severity of HSV epithelial keratitis.

### Silencing TRIM21 Controls HSV-1 Replication in HSV-1 Infected Corneas

**FIGURE 3:**
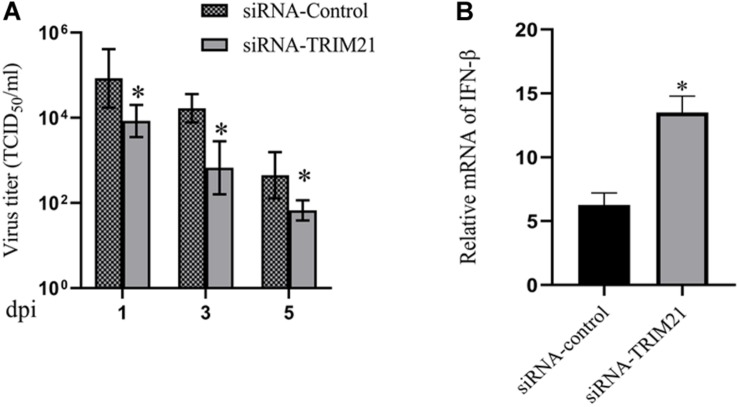
Silencing TRIM21 controls HSV-1 replication in HSV epithelial keratitis. **(A)** Viral titers in tear films from siRNA-TRIM21 and siRNA-control treatment mice were determined by TCID_50_ assay on days 1, 3, and 5 post-infection. **(B)** The relative mRNA level of IFN-β in corneas from each group measured by qPCR. *n* = 3 per group. Data are shown as mean ± SD of three independent experiments. ^∗^*P* < 0.05.

Tear samples from the siRNA-TRIM21 and siRNA-control treatment groups were subjected to TCID_50_ assay to investigate the effect of TRIM21 on virus replication. As shown in Figure 3A, silencing TRIM21 controlled the virus particle release at 1, 3, and 5 dpi significantly. Type I interferon signaling is identified as a critical step for controlling viral replication. Therefore, we further examined the transcript level of IFN-β in corneas at 3 dpi. The qPCR results showed that the IFN-β expression in the siRNA-TRIM21 treated corneas was approximately twice that of the siRNA-control treated corneas (Figure 3B).

### Silencing Trim21 Reduces Pro-inflammatory Cytokines in Hsv-1 Infected Corneas

**FIGURE 4:**
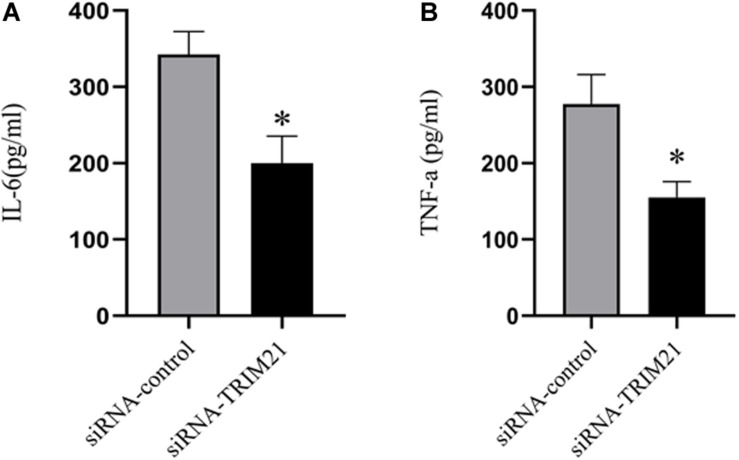
Silencing TRIM21 reduces pro-inflammatory cytokines in HSV epithelial keratitis. ELISA measured the protein expression levels of corneal IL-6 **(A)** and TNF-a **(B)**. *n* = 3 per group. Data are shown as mean ± SD of three independent experiments. ^∗^*P* < 0.05.

To determine the effects of TRIM21 on pro-inflammatory cytokines in HSV epithelial keratitis, three corneas from the siRNA-TRIM21 and siRNA-control treatment groups were pooled to obtain one sample for analysis of IL-6 and TNF-a expression levels by ELISA. As shown in Figure 4, the expression of IL-6 and TNF-a were both increased in HSV epithelial keratitis at 3 dpi. Compared with the siRNA-control treated corneas, the expression of these two pro-inflammatory cytokines in the siRNA-TRIM21 treated corneas was significantly suppressed.

### TRIM21 Inhibited STING-IRF3 Signaling in HSV-1 Infected HCE Cells

**FIGURE 5:**
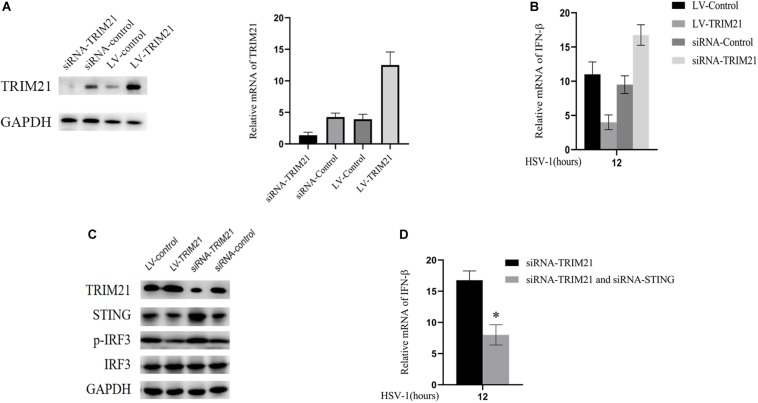
TRIM21 inhibited STING-IRF3 signaling in HSV-1-infected HCE cells. **(A)** The protein (left) and mRNA levels (right) of TRIM21 in HCE cells were assessed to confirm the transfection efficiency of lentivirus and siRNA before HSV-1 infection. **(B)** The mRNA level of IFN-β in each group was examined after HSV-1 infection 12 h. **(C)** The protein expression levels of TRIM21, STING, phosphorylation of IRF3, and IRF3 were examined after HSV-1 infection 12 h. **(D)** HCE cells pretreated with both siRNA-TRIM21 and siRNA-STING. After HSV-1 infection 12 h, qPCR detected the production of IFN-β. Data are shown as mean ± SD of three independent experiments. ^∗^*P* < 0.05.

Next, we explored the mechanism of TRIM21 affecting HSV-1 replication in human corneal epithelial cells (HCE cells). To interfere with TRIM21 expression, lentivirus-TRIM21 (LV-TRIM21), LV-control, siRNA-TRIM21, and siRNA-control were used to transfect HCE cells, respectively. First of all, transfection efficiency (Figure 5A) was assessed by the protein and mRNA levels of TRIM21 in HCE cells. Subsequently, HSV-1 infected the pretreated cells at MOI of 10. qPCR results showed that the expression of IFN-β was enhanced in siRNA-TRIM21 pretreated cells and suppressed in LV-TRIM21 pretreated cells (Figure 5B). In order to explore the mechanism of TRIM21 on the production of IFN-β in HSV-1 infected HCE cells, Western Blotting was used to detect the STING/IRF3 signaling pathway. As shown in Figure 5C, the expression of STING and phosphorylated IRF3 was enhanced in siRNA-TRIM21 pretreated cells after HSV-1 infection 12 h. Conversely, the expression of STING and phosphorylated IRF3 was reduced in LV-TRIM21 pretreated cells. When we pretreated HCE cells with both siRNA-TRIM21 and siRNA-STING, qPCR results showed that the level of IFN-β production decreased significantly compared to siRNA-TRIM21 pretreated cells alone (Figure 5D). The above results showed that in HSV-1 infected HCE cells, TRIM21 regulates the production of type I interferon by regulating the STING/IRF3 signaling pathway.

## Discussion

B30.2 domain in the TRIM21 protein structure can identify the invading virus so that TRIM21 plays a vital role in the process of virus infection. HSV-1 infection can occur anywhere in the eye, and the most common presentation is epithelial keratitis, which usually self-healing within a week after infection. However, the underlying mechanism of TRIM21 in HSV epithelial keratitis remains unclear. Our study demonstrated that TRIM21 is abnormally high expressed in HSV epithelial keratitis and aggravates the severity of epithelial keratitis by promoting type I interferon signaling.

Previous studies have shown that TRIMs protein can be expressed in most normal tissues (Hatakeyama, 2017). This study is the first to confirm that TRIM21 is constitutively expressed in the cornea of C57BL/6J mice without pathogen infection. The initial manifestation of HSV epithelial keratitis is punctate keratopathy. As the virus spreads uncontrollably, the disease progresses from punctate keratitis to dendritic keratitis or a geographic ulcer. HSV-1 McKrae strain (10^6.6^ TCID_50/_ml) was applied to the right cornea of C57BL/6J mice. We found that the expression of TRIM21 in corneas was abnormally increased at 2 and 4 days post HSV-1 infection (dpi). Immunofluorescence results revealed that, at 3 dpi, TRIM21 was located in the cytoplasm of corneal epithelial cells and compared with the normal cornea, its expression was enhanced with strong fluorescence. These results suggest that TRIM21 is involved in the development of HSV epithelial keratitis.

To further ascertain the role of TRIM21 in HSV epithelial keratitis, siRNA was used to limit TRIM21 expression in the corneas before establishing the HSV epithelial keratitis mice model. Through the clinical scores and histopathology examination, we found that TRIM21 can effectively reduce the severity of HSV epithelial keratitis. Previous research has shown that the severity of HSV epithelial keratitis is related to the virus clearance rate (Babu et al., 1996; Twardy et al., 2011). In this study, silencing TRIM21 controlled the virus particle release at 1, 3, and 5 dpi significantly, indicating that silencing TRIM21 increased the virus clearance rate. Once HSV-1 infects corneal epithelial cells, recognition of viral ligands by pattern recognition receptors (PRRs) in host cells will elevate the transcription of various interleukins and tissue necrosis factor to clear the virus (Akira et al., 2001; Akira and Takeda, 2004). At the same time, the inflammatory cascade induces an excessive immune response leading to progressive corneal opacification. At 3 dpi, we found that the expression of pro-inflammatory cytokines (IL-6 and TNF-a) in the siRNA-TRIM21 treated corneas was significantly repressed. Consistent with previous reports, IL-6 and TNF-a are the major drivers of corneal inflammation, and various strategies to block these cytokines have proven effective in decreasing the severity of HSV epithelial keratitis in mice (Rajasagi et al., 2017).

Cytosolic DNA sensors are the most recently described class of PRRs, which include cGAS (Li et al., 2013), AIM2 (Fernandes-Alnemri et al., 2009), DAI (Takaoka et al., 2007), DDX41 (Zhang et al., 2011) and IFI16 (Unterholzner et al., 2010). Viral nucleic acids of HSV-1, recognized by various PRRs, can act as activators of type I interferons signaling pathways so that promote antiviral immune responses. Several studies have identified that the antiviral response mediated by type I interferon is a key way to limit viral replication (Gresser et al., 1979; Zawatzky et al., 1982; Ellermann-Eriksen et al., 1986). In the absence of type I interferon signaling (interferon receptor A1 knocked out and CD118 −/−), the virus replication ability was enhanced, and the mortality of mice was increased, which highlighted the crucial defensive role of type I interferon in the control of HSV-1 infection (Leib et al., 1999; Luker et al., 2003; Conrady et al., 2011a). In the research of CVB3 and HBV, TRIM21 functions as a positive regulator of type I interferon, promoting a comprehensive antiviral response (Liu et al., 2018; Zhang et al., 2018). However, in the research of JEV infection, TRIM21 functions as a negative regulator of type I interferon, promoting JEV virus replication (Manocha et al., 2014). In our study, qPCR results showed that silencing TRIM21 enhanced type I interferon signaling at 3 days post HSV-1 infection.

The stimulator of interferon genes (STING) acts as a converging point of cytosolic DNA receptors to promote signal transmission to downstream effector IRF3, leading to the expression of type I interferon (Jiang et al., 2017). In order to survive in host cells, HSV-1 encodes a variety of proteins that inhibit STING-IRF3 signaling to prevent host cells from producing type I interferon (Christensen et al., 2016). It means that STING-IRF3 signaling is critical in host defense against the virus (Abe et al., 2013; Barber, 2014). In the experiment of HCE cells infected with HSV-1, we found that up-regulating TRIM21 inhibited STING-IRF3 signaling and reduced IFN-β expression, while silencing TRIM21 enhanced STING-IRF3 signaling and increased IFN-β expression. HCE cells were afterward pretreated with siRNA to silence both TRIM21 and STING at the same time. We found that the expression of IFN-β relatively decreased compared to the siRNA-TRIM21 treated alone. Thus, these results show that the effect of TRIM21 on type I interferon is mediated by STING-IRF3 signaling.

Collectively, our results have identified conclusions as follows. TRIM21 is constitutively expressed in normal corneas and is abnormally high expressed in HSV epithelial keratitis. Highly expressed TRIM21 enhances the replication of HSV-1 in corneal epithelial cells via suppressing the production of type I IFN by inhibiting STING/IRF3 signaling. It also promotes the production of pro-inflammatory cytokines IL-6 and TNF-a to the point of synergistically exacerbating the severity of HSV epithelial keratitis.

## Data Availability Statement

The data used to support the findings of this study are available from the corresponding author upon request.

## Ethics Statement

The animal study was reviewed and approved by the Ethics Committee of Shengjing Hospital, China Medical University (Ethics Number: 2018PS128K).

## Author Contributions

LX and TT contributed to the design of the experiments. TT contributed to the conduction of the experiments and editing of the manuscript.

## Conflict of Interest

The authors declare that the research was conducted in the absence of any commercial or financial relationships that could be construed as a potential conflict of interest.
